# 1. The Data Librarian: laboratories today—the need for the Librarian

**DOI:** 10.1155/S1463924697000230

**Published:** 1997

**Authors:** Joe Liscouski

**Affiliations:** LASF PO Box 38 Groton MA 01450 USA

## Abstract

This first part of ‘The Data Librarian’ describes the current situation in analytical laboratories and the need for the Data Librarian. The second part of the paper (Liscouski, J., 1997, Journal of Automatic Chemistry, 19, 199-204) will examine the features of the Data Librarian.


Journal of Automatic Chemistry, Vol. 19, No. 6 (November-December 1997) pp. 193-197

### 1. The Data Librarian: laboratories today—the need for the Librarian

**Joe Liscouski**
*LASF, PO Box 38, Groton, MA01450, USA*

*This first part of 'The Data Librarian' describes the current situation in analytical laboratories and the need for the Data Librarian. The second part of the paper* (Liscouski, J., 1997, *Journal of Automatic Chemistry,* **19**, 199–204) *will examine the features of the Data Librarian.*

### Why is the Data Librarian needed?

Scott Mutter is an artist who specializes in illustrations that mix images and challenge perceptions. One of them is a picture that shows a cityscape resting on the card catalogue of a large library—situated so that the library appears to be the structural foundation of the city. The caption reads: 'A culture and what it produces is made possible and is reflective of the knowledge that underlies it'. We build our societies on knowledge.

If that is true of society, it is more so of business. The organizations, buildings, factories, and research facilities are just the current implementation of knowledge and learning. As we learn new things, corporations change. Production facilities are modified to reflect research on the nature of things and how to make them. Laboratory work generates new knowledge, understandings and technologies, and those technologies produce new products. The real corporate assets consist of knowledge, information and data.

Companies generally have been poor stewards of their laboratory data.[1] That problem is not a matter of training people, but a result of laboratory computing systems that generated hundreds and thousands of files per day using computer systems that are designed to manage programs and not data. It is a problem that is not unique to the laboratory but common throughout industry, affecting office workers, as well as scientists and technicians. Microsoft, in version 5 of Word (its word processing software) appears to recognize the problem of managing documents. It has provided a crude document management facility (see figure 1) which allows a user to give documents a title, subject, author, version and keywords.

These attributes can be used to search the document database through a 'find file' function. While this can be a usable tool for an individual user, it does not support shared access, is limited to the local hard disk, does not provide any security or backup or file migration, and can be easily fooled into providing the wrong material. It does show, however, that there is a real problem, and that problem needs to be addressed with a well thought through system. A robust solution instead of a band-aid.

The Data Librarian is needed to provide a long-term, rational approach to managing data files for maintaining the value and accessibility of data long-term when those data are critical to a company's success and very survival. What would happen to your companies if critical legal and financial records could not be found? The problem is far from trivial, as noted by Jeff Rothenberg in a recent article in *Scientific American* (Vol. 272, No. 1):

> *There have already been several potential disasters. A 1990 House of Representatives report describes the narrow escape of the 1960 U.S. Census data. The tabulations were originally stored on tapes that became obsolete faster than expected as revised recording formats supplanted existing ones (although most of the information was successfully transferred to newer media). The report notes other close calls as well, involving tapes of the Department of Health and Human Services; files from the National Commission on Marijuana and Drug Abuse, the Public Land Law Review Commission and other agencies; the Combat Area Casualty file containing P.O.W and M.I.A. records for the Vietnam War; and herbicide information needed to analyse the impact of Agent Orange. Scientific data are in similar jeopardy, as irreplaceable records of numerous experiments conducted by the National Aeronautics and Space Administration and other organizations age into oblivion.*

In testing laboratories governed by the US's Food and Drug Administration, not being able to find critical data can be the cause for being shut-down—that can mean that an entire plant's current production of a pharmaceutical product could be lost. The same is true for labs that have to meet the requirments of the Environmental Protection Agency. Less disastrous, but just as serious, is lost time in searching for data, and repeating tests, which build in delays and cost into products. These are problems that the Data Librarian is designed to solve.

### Why is the Data Librarian important to scientific work?

Science needs the Data Librarian for two reasons:

(1) The need is acute and growing due to regulatory and legal pressures.
(2) Scientific and laboratory applications of computing have been in the forefront of technology development. That is true here as well. The scientific work in collaborative computing, data sharing, electronic

[1] Laboratory data is usually recorded in notebooks, checked and signed. The real problems occur for long-term data access, management, and utilization. Active data—material that is being used in the current project—may be easy to account for. As people change jobs or responsibilities and their memory of data and its location fade, as data are moved to archives or away from active, immediate use, the ability to find and use data decreases.

0142-0453/97 $12·00 © 1997 Taylor & Francis Ltd

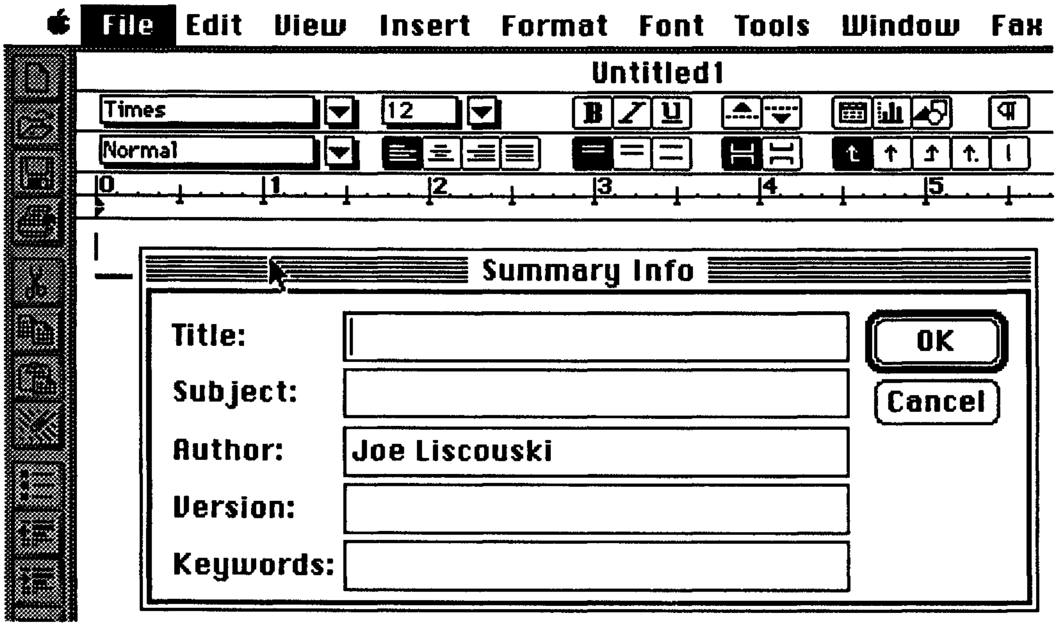

*Figure 1. Document management with Word 5.*

notebooks, etc. is a forerunner of the products that will become commonplace in office and educational work.

The use of computing in laboratory and scientific work has suffered from the lack of standards and planned basis for systems integration. Each software system is viewed as an entity unto itself, with co-operation between vendors and systems—readily found in the commercial office automation market—lacking. The demand for a change in that behaviour is increasing. Companies are recognizing that laboratory data are their lifeblood and they need to maintain better access and utilization.

This paper describes how laboratory work is done today, and how it would change with the development of the Data Librarian.

### The current situation

Due to the current design of laboratory data systems, laboratory computing is divided into two layers: (1) the data station; (2) the Laboratory Information Management System (LIMS), which is an administrative tool for work scheduling, tracking test results, determining backlogs, and producing reports (figure 2). This layering is the result of product evolution.

Data systems came first, as an instrument vendor's response to customer needs and competitive pressures in working with instrument data and increased sample load. Perkin-Elmer, an instrument vendor who also owned a computer company, recognized a gap in laboratory management. 'Data' was handled by the data systems; laboratory 'information' (viewed as administrative material and the results of analysed data) needed another software system and so the LIMS was born.

The data system is responsible for taking signals or data from an instrument, analysing them, calculating percentage composition, and then storing the data and results. The most common laboratory instrument used in automated systems is the chromatograph which is used to separate mixtures of materials into their components and then produce a signal (a peak) proportional to the amount of each component. The data system acquires the signal, determines the size of the peaks in the sample under test, compares it to peaks of known amounts and then calculates the amount in the sample. These systems are run by a single software program usually on an IBM PC compatible computer. The data and calculated results are stored within this program's data structures. The results can be sent to a printer or stored in an external file. Until 1992 the data were maintained in a proprietary format which was different for each vendor. With the release of the AIA specification, vendors could provide the data in an industry standard format.

The LIMS is the job scheduler for a laboratory. Samples submitted for testing are logged into the LIMS, and scheduled. Test results are entered (either manually or electronically) as they are produced and, when a sample is done, a report is printed. Electronic entry of data is highly desirable since it reduces the workload on people and eliminates transcription errors.

### Working with the Data Librarian

There are short- and long-term design issues resulting from the introduction of the Data Librarian.

*Short-term:* Data systems will remain much as they are today but their role has shifted. They are no longer the primary data storage manager. Their role is to acquire

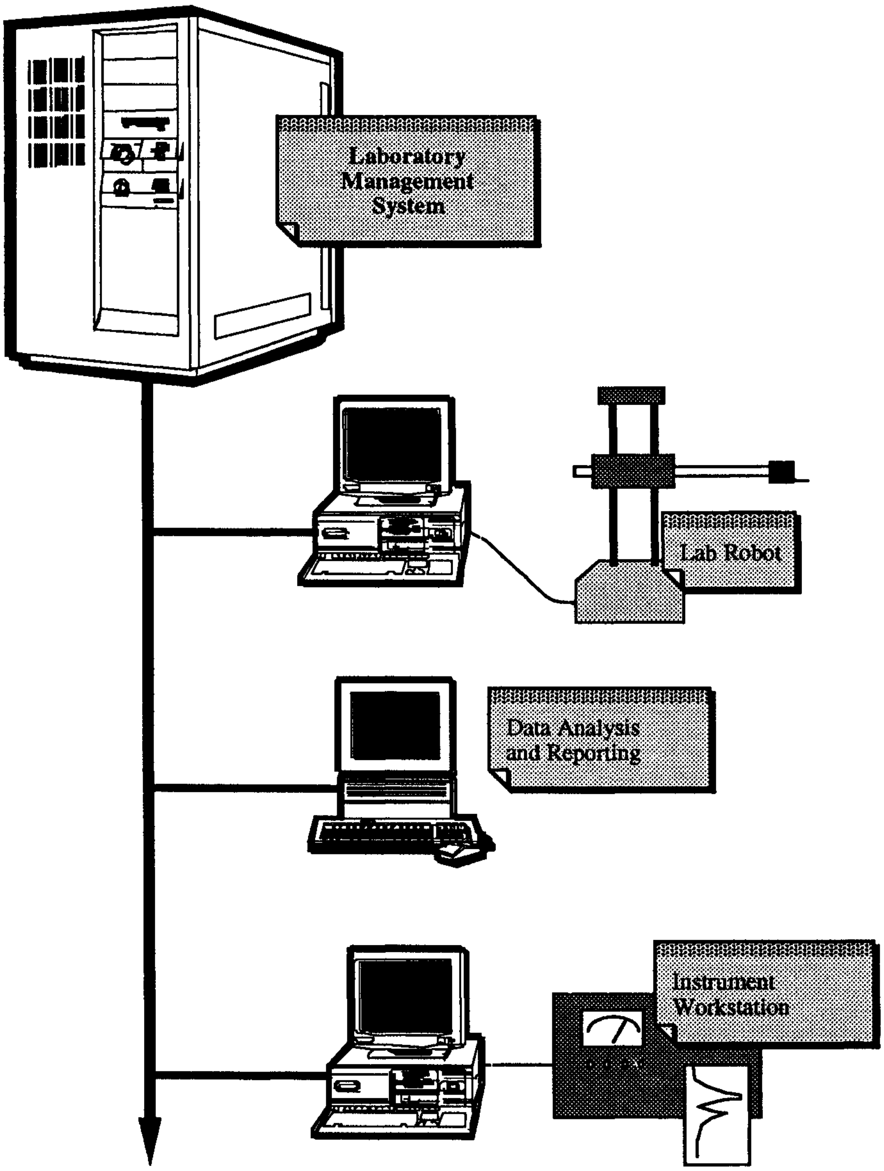

*Figure 2. LIMS.*

data, analyse them, report, and then export the data to the Data Librarian for use by other software and long-term storage.

A relatively new market for software has begun to emerge for data analysis software as a result of the 1992 AIA Chromatography Data Format Standard. Several vendors are developing programs that analyse previously acquired data, using the standardized data format as an input. (These include Separation Systems (Gulf Breeze, Florida) which is offering a simulated distillation calculation and reporting package; H&A Scientific (Greenville, NC) which is developing chromatography support packages; and WindowChem Software which is offering the AIA Explorer and AIA Browser for examining data in the AIA standard format.) This is significant and precisely what is expected by the LASF laboratory model which was first made public in 1992 and is a major point in *Laboratory and Scientific Computing: a Strategic Approach* (figure 3).

Prior to this development, someone wanting a new approach to analysing instrument data would have to either convince an existing instrument vendor to incorporate his or her algorithms into their product, or, create his or her own system for acqusition, analysis, storage, and reporting; the problem that needs to be overcome is access to the primary instrument data—now solved by the data format standards.

The Data Librarian provides the basis for organizing laboratory data, and, a foundation from which stand-alone analysis and reporting systems can access data—solving the second point above. This removes the limitation mentioned in the first issue in table 1.

The data files managed by the Librarian are referenced by file name, as well as descriptive attributes, such as sample ID, analysis type, standardized naming convention, product, data of production, analysis date, product lot numbers, etc. This multi-key referencing means that it is easy to find data based on material and analysis

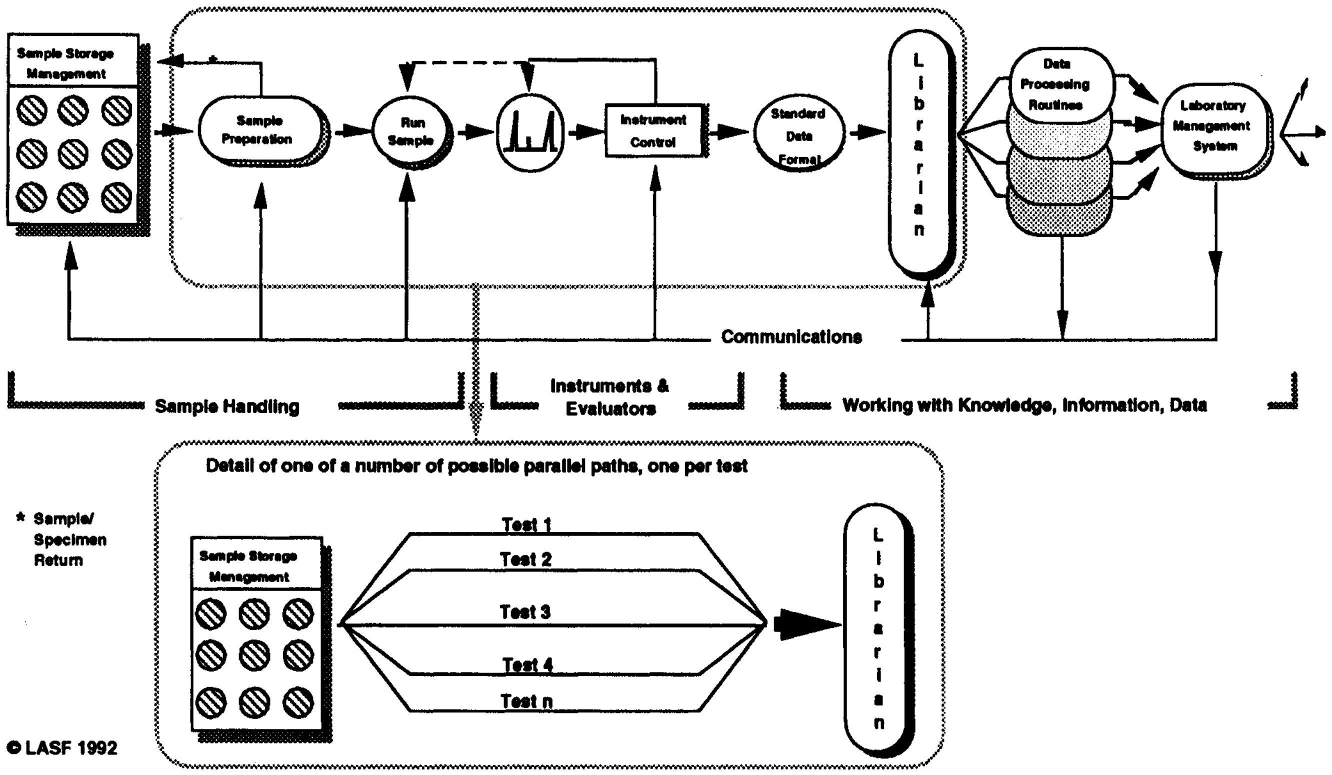

*Figure 3. Proposed LASF laboratory models.*

*Table 1. Problems inherent in LIMS.*

| *Issue* | *Ramifications* |
|---|---|
| 1. The ability to work with laboratory data is limited by the data system vendors | Since the data systems contain all functions for controlling the instruments, acquiring the data, storing it, analysing it, and producing reports, the laboratory's ability to work is limited by the data systems software vendor. This is analogous to having the word processor, or spreadsheet vendor owning and controlling your text and data; limiting your ability to work by determining what functions they would add. The inability to export it in a format that could be read by other software means that once you were a Microsoft Word user, you were always a Word user—you couldn't change word processors because you couldn't use the text anywhere else. |
| | Note: the development of data format standards is now (only in the last three years) allowing people to export the data into individual files readable by most vendor products. |
| 2. New data format standards will cause large numbers of files to be created | The movement to data format standards is going to create a large number of files that have to be managed by the laboratory. Laboratory systems are not designed to be effective file managers. Data systems will manage data within their own file structure, but once it is exported—either because the user wants to work with it outside the data system, or the system is becoming full and the contents need to be archived—the users are on their own. Data files will be identified by a short file name, and the possibility exist for data loss through inadequate cataloguing, deletion, improper backup, file replacement, or mismanagement. |
| 3. The lack of a file manager makes it difficult to find key data when they are needed | If people need a particular data file, particularly if it is historical data, it may take hours to find—in a regulated environment that can bring a citation from a government inspector. Data may be spread over several systems, may be on archive tapes which are manually managed and catalogued, errors can occur and media can be corrupted. This problem builds time and cost into projects and prevents companies from getting the full benefit from their investment in research and testing—data that cannot be found, cannot be used and may as well not exist! That is a poor return on laboratory investments. |
| | The problem is compounded when someone wants to run a study of historical data on a set of samples. Each sample may have data in several files, all of which have to be found one at a time. This can prevent useful research from being done. It may also cause patents to be denied or the loss of a product liability lawsuit. |
| 4. Linking data systems and LIMS is a custom design effort | The property focus of systems vendors increased the cost of linking the two major components of lab systems. Those vendors who offer both provide built in links. LIMS only vendors have taken either or both of two courses: (1) built in functions that incorporate simple ASCII files (contain the reduced results); and (2) built in proprietary interfaces for lab instruments. |

characteristics. Historical studies are simpler, because the data are accessible, improving the value of the data through increased utilization and the return on investments in laboratory automation.

The development of stand-alone analysis and reporting software should foster the development of standardized methods of communicating analytical results to the LIMS. This will greatly improve the ability of laboratories to integrate the LIMS into the rest of the lab's data and information stream.

*Longer term benefits:* Further along in time, the original two-layered model will collapse with the functions now in data systems being divided into separate modules for acqusition, direct storage into the Librarian, and then using stand-alone post-run processing and reporting systems.

Data format standards are the basis for a potential revolution in laboratory automation. Without the organizing functions of the Librarian (which is currently a concept and not a product), that revolution may lead to chaos and having each lab develop their own response to the problem. The Librarian is a unifying factor, and will provide a solid basis for turning the promise of data format standards into a functioning reality.

The problems with this design are shown in table 1.

*Part 2 of this two-part paper will describe the introduction of the Data Librarian.*

